# Sero-prevalence of anti-Leptospira antibodies and associated risk factors in rural Rwanda: A cross-sectional study

**DOI:** 10.1371/journal.pntd.0009708

**Published:** 2021-12-07

**Authors:** Etienne Ntabanganyimana, Robert Giraneza, Vincent Dusabejambo, Appolinaire Bizimana, Camila Hamond, Augustin Iyamuremye, Placide Nshizirungu, Raphael Uzabakiriho, Marc Munyengabe, Elsio A. Wunder, Cameron Page

**Affiliations:** 1 College of Medicine and Health Sciences, University of Rwanda, Kigali, Rwanda; 2 Gihundwe District Hospital, Rusizi, Rwanda; 3 Centre Hospitalier Universitaire de Butare, Huye, Rwanda; 4 College of Business and Economics, University of Rwanda, Kigali, Rwanda; 5 Department of Epidemiology of Microbial Diseases, Yale School of Public Health, New Haven, Connecticut, United States of America; 6 Ruhango Referral Hospital, Ruhango, Rwanda; 7 Gonçalo Moniz Institute, Oswaldo Cruz Foundation, Brazilian Ministry of Health, Salvador, Brazil; 8 University Hospital of Brooklyn, SUNY Downstate Medical Center, New York City, New York, United States of America; Medical College of Wisconsin, UNITED STATES

## Abstract

**Background:**

Leptospirosis is a zoonotic disease transmitted through the urine of wild and domestic animals, and is responsible for over 50,000 deaths each year. In East Africa, prevalence varies greatly, from as low as 7% in Kenya to 37% in Somalia. Transmission epidemiology also varies around the world, with research in Nicaragua showing that rodents are the most clinically important, while studies in Egypt and Chile suggest that dogs may play a more important role. There are no published studies of leptospirosis in Rwanda.

**Methods & findings:**

We performed a cross-sectional survey of asymptomatic adults recruited from five occupational categories. Serum samples were tested using ELISA and Microscopic Agglutination Test (MAT). We found that 40.1% (151/377) of asymptomatic adults had been exposed to *Leptospira* spp. Almost 36.3% of positive subjects reported contact with rats (137/377) which represent 90.7% among positive leptospira serology compared with 48.2% of negative subjects (182/377) which represent 80.5% among negative leptospira serology (OR 2.37, CI 1.25–4.49) and 1.7 fold on prevalence ratio and 2.37 of odd ratio. Furthermore, being a crop farmer was significantly associated with leptospirosis (OR 2.06, CI 1.29–3.28). We identified 6 asymptomatic subjects (1.6%) who met criteria for acute infection.

**Conclusions:**

This study demonstrates a high prevalence of leptospiral antibodies infection among asymptomatic adults in rural Rwanda, particularly relative to neighboring countries. Although positive subjects were more likely to report rat contact, we found no independent association between rats and leptospirosis infection. Nonetheless, exposure was high among crop farmers, which is supportive of the hypothesis that rats together with domestic livestock might contribute to the transmission.

Further studies are needed to understand infecting Leptospira servers and elucidate the transmission epidemiology in Rwanda and identify means of host transmitters.

## Author summary

Leptospirosis is the most widespread zoonosis in the world, and infections are underappreciated in regions of the world where it may be mistaken for malaria and other tropical illnesses. We performed a seroprevalence study of leptospirosis in healthy asymptomatic adults in Rwanda. Among 377 subjects, we found evidence of infection in 40.1% (151 / 377), which is higher than in neighboring countries such as Kenya (7 to 16%), Tanzania (10 to 15.8%) and Uganda (15%). Our data suggest that leptospirosis may be a common cause of febrile illness in Rwanda, but further studies are needed to differentiate active from asymptomatic.

## Introduction

Leptospirosis is a life-threatening environmentally-transmitted disease. The disease has a worldwide distribution, with prevalence being ten times higher in tropical than temperate areas [1]. In 2015, a systematic review identified 80 studies meeting high- and medium-quality criteria from 34 countries [2]. This review estimated an incidence of 1.03 million cases annually and 58,900 deaths. Previous research has found that leptospirosis infection is associated with poverty, including low education, poor housing, poor hygiene, low income and working as a farmer [3]. Leptospirosis is a zoonotic infection caused by pathogenic spirochetes of the genus *Leptospira*. The bacteria multiplies in the renal tubules of rodents, dogs, cows, and other domestic and wild mammals, and then shed in the environment through their urine [4]. Humans are accidental hosts, and the transmission of the disease is usually higher during rainfall period in tropical countries [5] and areas where social inequality is a problem. *Leptospira* enters the body through mucous membranes, conjunctivae, or small abrasions, and quickly disseminates and multiply in all organs. The infection in humans can vary from asymptomatic to an acute disease like Weil’s syndrome, characterized by liver and renal failure, and pulmonary haemorrhage syndrome (LPHS). If not diagnosed and treated in time, lethality varies from 10–50% [4].

The gold standard assay for diagnosis of leptospirosis is the Microscopic Agglutination Test (MAT) [6]. This assay requires live cultures and is somewhat labor intensive with specific skills required. In low-resouce settings, leptospirosis antibodies can be detected using enzyme-linked immunosorbent assay (ELISA) in serum, with the understanding that Lepto-ELISA have low sensitivity and specificity [7]. Traditional risk factors associated with leptospirosis have been occupational, especially in rural areas.

However, there has been an increasing awareness of the disease as a cause of outbreaks during sporting events, natural disasters and travelers. Furthermore, with globalization and migration, the disease has become a major burden in urban areas of resource-poor countries and among subsistence farmers. Transmission epidemiology varies around the world, but rodents are generally the main reservoir, especially in urban areas, with dogs and domestic livestock also playing a role [8,9]. Prevalence and incidence data from Africa is still scarce. In Sub-Saharan Africa, there are a number of factors that put the population at increased risk of leptospirosis infection, including urban population density, poor infrastructure to manage flooding [10]. In November 2005, a cross-sectional study conducted in Tanzania, which borders Rwanda, showed a seroprevalence of 15% in 199 healthy participants [11]. The most recents studies which were conducted in Tanzania reported 10% leptospiral antibodies prevalence in slaughterhouse workers in Mwanza [12] and 15.8% antibodies prevalence in sugarcane plantation and fishing communities in Kagera region [13]. A study done in two parts of Kenya, which is in the same region of East Africa as Rwanda, found a prevalence of 16.9% among 130 asymptomatic adults in the coast province, and 7.4% among 353 healthy people in Nyanza province (near Lake Victoria) [7].

To determine the burden of leptospirosis in Rwanda, we performed a study of asymptomatic adults from a variety of occupations living in two different regions of the country.

## Objectives

### Specific objective 1

To determine the prevalence of *Leptospira* in Rwanda.

### Specific objective 2

To determine which exposures and risk factors are associated with Leptospirosis infection in Rwanda.

### Hypothesis 1

The asymptomatic population of Rwanda has a moderate to high prevalence of prior infection with leptospirosis.

### Hypothesis 2

Among asymptomatic subjects in Rwanda, previous infection with Leptospirosis correlates with established risk factors.

## Methods

### Ethics statement

The Institutional Review Board (IRB) of College of Medicine and Health Sciences (CMHS), University of Rwanda (UR) approved the study (Ref: CMHS/IRB/277/2015). All subjects were interviewed in their local language (Kinyarwanda), were verbally informed about the purpose of the study, and gave oral consent to participate. Subjects were also given a paper consent form, written in their local language, to read and sign.

### Study design and methodology

Rwanda is a small, land-locked country of 12 million people in East Africa, bordering Tanzania, Uganda, Burundi, and the Democratic Republic of Congo The country comprises an area of 26,000 square kilometers, roughly the size of the U.S. state of Maryland [14]. Rwanda is one of Africa’s most densely populated countries, and 75% of its residents engage in subsistence agriculture [14]. By far one of the most commonly grown crops in Gisagara district is rice which is cultivated on 2361 hectares with 7593 farmers including 1970 females and 5623 males according to Gisagara district, agriculture unit. With a 2020 GDP per capita of $ 797.856, Rwanda is classified as a low-income country [15]. Gisagara district is one of the districts with the highest percentage of poor (27%) and extremely-poor (32%) residents, which compares unfavorably to national average of 24% and 21%, respectively [16].

We performed a cross-sectional study of asymptomatic individuals from Gisagara and Huye districts in the Southern Province of Rwanda. To be eligible for inclusion in the study, subjects had to be 21 years of age or older and have no current medical complaints. We evaluated all potential subjects equally and included every subject who met eligibility. We took a set of vital signs for every participant, and candidates were excluded if they were found to have a temperature of 38° C or above.

During two weeks in January 2016, we recruited subjects from 5 occupations. Four were “high-risk” occupations which have been associated with leptospirosis in prior studies [11]: slaughterhouse workers, cattle farmers, crop farmers, and fish farmers. The fifth “low-risk” occupation was University medical students, which were used as a baseline group. Data was collected from all recruited subjects about risk factors for leptospirosis infection.

### Blood sampling and serological screening

377 participants met inclusion criteria, sampled and provided consent to collect their demographic data and whole blood was drawn from each and transported immediately to the Serology Unit at the University Hospital of Butare, where the serum was prepared and stored at -70°C. Once all samples were obtained, ELISA IgG and IgM testing was performed in Rwanda at the Centre Hospitalier Universitaire de Butare according to the manufacturer instruction. After ELISA testing was complete, the Microscopic Agglutination Test (MAT) was performed in USA at Yale School of Public Health in accordance with previous published protocols: Goris and Hartskeerl (Curr. Protoc. Microbiol. 32:12E.5.1-12E.5.18). For the MAT, thirty reference strains were used, representing 10 pathogenic species and one saprophytic species (Table 1). Leptospires were cultivated in liquid EMJH medium (Johmson et al, 1967) supplemented with 1% rabbit serum. The cultures were incubated up to 7 days at 29°C, till they reached log phase (between 4–5 days of incubation). The serovar representing the latter one, Patoc, was used as a marker for possible infections with serovars not included in the panel. Sera samples with antibodies that reacted only to Patoc could indicate unspecific reactions of anti-leptospiral anitbodies generated to a serogroup or serovar not included in the panel. The screening assay started at 1:100 dilution and all positives were titred (S1 Table).

**Table 1:** Leptospira reference strains used for serum of asymptomatic Rwanda adult participants.

Species	Serogroup	Serovar	Strain
*L*. *alexanderi*	Manhao	Manhoa 3	L 60T
*L*. *alstoni*	Ranarum	Pingchang	80-412T
*L*. *borgpetersenii*	Ballum	Ballum	Mus 127
*L*. *borgpetersenii*	Mini	Mini	Sari
*L*. *borgpetersenii*	Tarassovi	Tarassovi	Perepelitsin
*L*. *borgpetersenii*	Ballum	Castellonis	Castellon 3
*L*. *interrogans*	Autumnalis	Autumnalis	Akiyami A
*L*. *interrogans*	Bataviae	Bataviae	Van Tienen
*L*. *interrogans*	Canicola	Canicola	H. Ultrecht IV
*L*. *interrogans*	Djasiman	Djasiman	Djasiman
*L*. *interrogans*	Hebdomadis	Hebdomadis	Hebdomadis
*L*. *interrogans*	Icterohaemorrhagiae	Icterohaemorrhagiae	RGA
*L*. *interrogans*	Icterohaemorrhagiae	Copenhageni	M 20
*L*. *interrogans*	Pomona	Pomona	Pomona
*L*. *interrogans*	Sejroe	Hardjo	Hardjoprajitno
*L*. *interrogans*	Australis	Bratislava	Jez Bratislava
*L*. *interrogans*	Sejroe	Wolffi	3705
*L*. *interrogans*	Pyrogenes	Pyrogenes	Salinem
*L*. *interrogans*	Icterohaemorrhagiae	Copenhageni	L1 130
*L*. *interrogans*	Pyrogenes	Manilae	L495
*L*. *kirschneri*	Cynopteri	Cynopteri	3522C
*L*. *kirschneri*	Grippotyphosa	Grippotyphosa	Duyster
*L*. *kmetyi*	Tarassovi	Malaysia	Bejo-Iso9
L. mayottensis	ND	ND	200901122
*L*. *noguchii*	Louisiana	Louisiana	LSU 1945
*L*. *noguchii*	Panama	Panama	CZ 214 K
*L*. *santarosai*	Shermani	Shermani	1342 K
*L*. *weilii*	Celledoni	Celledoni	Celledoni
*L*. *weilii*	Javanica	Coxi	Cox
*L*. *biflexa*	Semaranga	Patoc	Patoc 1

Subjects that tested positive by Microscopic Agglutination Test (MAT) for Leptospiral antibodies, and their serum titer response to each of the 30 reference strains

Source: Authors’Compilation, 2016

**Fig 1:**
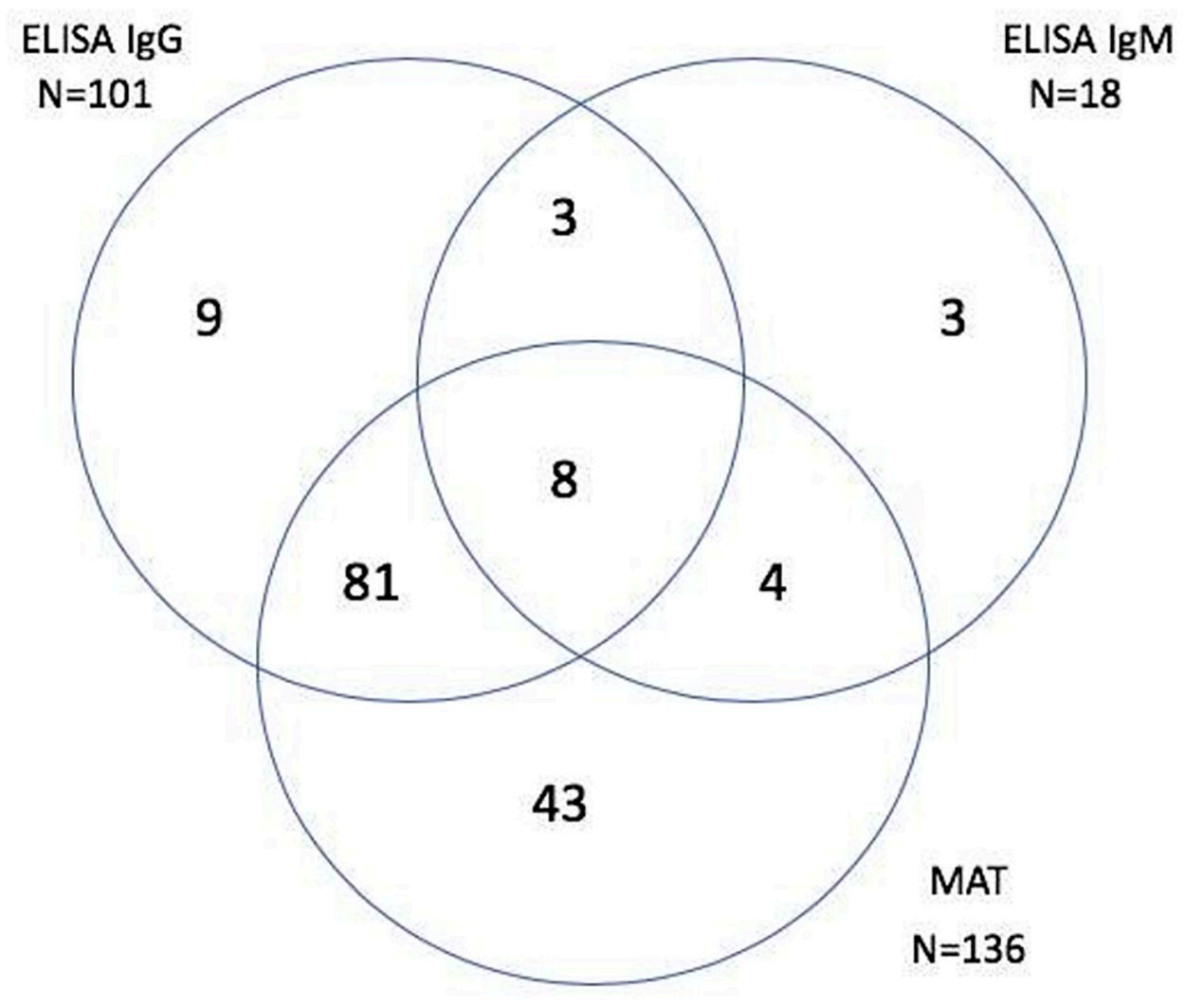
Venn diagram of the distribution of leptospirosis positive serum samples based on the three laboratory testing modalities that were used: MAT, ELISA IgG, and ELISA IgM. There were 151 total positive samples by any method.

The presumptive infecting serogroup was determined based on the serovar which the highest agglutination titer was detected, the samples were titrated up to 1:12,800. Based on the three laboratory testing modalities, there were 151 total positive samples by ELISA IgG, ELISA IgM and MAT (Fig 1).

For the ELISA assay, we used Leptospira ELISA tests from Diagnostic Automation/Cortez Diagnostic Inc. for both IgG and IgM anti-Leptospira antibodies The assay was performed based on instructions per manufacturer, and the results were reported as positive when OD was ≥1.0 and negative when OD was <1.0.

### Case definition

Each sample was tested with two assays, MAT and ELISA. The ELISA assay tested for both IgG and IgM anti-*Leptospira* antibodies. Subjects were classified as positive if they met any of the three following criteria: 1) ELISA IgG OD > 1.0, 2) ELISA IgM OD > 1.0, and/or 3) MAT ≥ 1:100.

### Statistical analysis

The multivariate logistic regression analysis between Leptospira serology as dependent variable and demographic features, animal exposure, and non-animal exposures of asymptomatic Rwandan adult participants. The use of multivariate was to measure the effect with consideration of auto-correlation where it existed. In the presentation of findings, the prevalence of out of total number of participants (n = 377), coefficient, p-value and OR [95% C.I]. This analysis was done in combination with the calculation of the prevalence of independence variables within dependent variable (the status of serology: Positive or negative). To perform these calculations, the statistical package for social science (SPSS) was used.

## Results

### Serology

**Fig 2:**
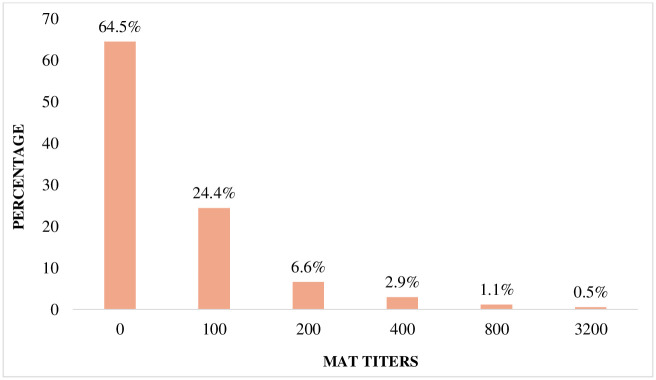
Bar chart of the distribution of leptospirosis MAT titers in serum samples collected from 377 asymptomatic adults in rural Rwanda. Results ranged from 0 to 3200 with the majority (64.5%) being undetectable. MAT: Microscopic Agglutination Test.

**Fig 3:**
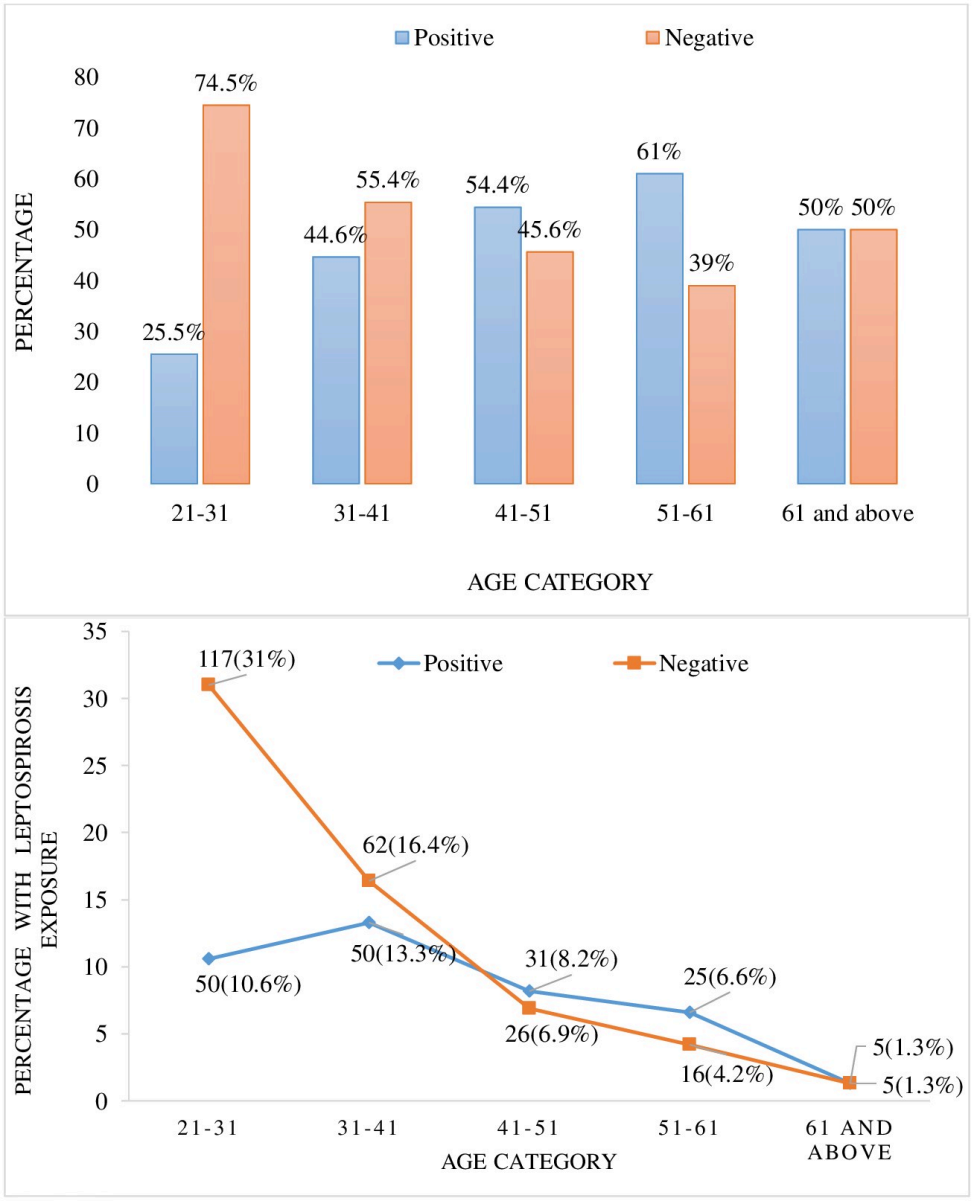
Prevalence of leptospirosis by age group among asymptomatic adults in rural Rwanda. The majority of unexposed subjects (52%) were age 21–31. Percentages are out of total positive or total negative.

Overall, 151 of 377 subjects (40.1%) had evidence of leptospirosis infection using at least one of the two assays. Of those, 101/377 (26.7%) were ELISA IgG positive, 18/377 (4.7%) were ELISA IgM positive, and 136/377 (36.0%) were MAT positive (Fig 1). A majority of the subjects that were positive by MAT (94/377; 24.9%) had the lowest titer, which was 1:100. The lack of paired sera made it impossible to determine what proportion of our positive cases represented a recent infection. However, among the MAT positive subjects, six had a titer ≥ 1:800, which is indicative of a recent exposure (Fig 2). Serogroup Icterohaemorrhagiae was the most prevalent among the positive samples determined by MAT (n = 78/377, 20.8%), with no other predominating Serogroup (Table 1). the highest prevalence of Leptospirosis infection by age group was 51–61 years old (Fig 3) and none of non-animal exposure showed significant association with leptospirosis infection (Table 2).

**Table 2:** Multivariate Analysis for the likelihood of non-animal exposures and *Leptospira* infection among asymptomatic adults in Rwanda.

	Prevalence out total number of participants (n = 377)		B	p-value	OR [95% C.I]
	Positive (40.1%)	Negative (59.9%)			
Intercept			-0.816	0.333	
Unpasteurized milk					
Yes	58(15.4%)	70(18.6%)	0.001	0.996	1.001[0.601; 1.668]
No	93(24.7%)	156(41.4%)	0^a^	.	.
Pasteurized milk					
Yes	43(11.4%)	91(24.1%)	0.362	0.281	1.437[0.743; 2.778]
No	108(28.6%)	135(35.8%)	0^a^	.	.
Boiled milk					
Yes	84(22.3%)	141(37.4%)	-0.062	0.830	0.940[0.532; 1.660]
No	67(17.8%)	85(22.5%)	0^a^	.	.
Boiled water					
Yes	70(18.6%)	132(35%)	0.149	0.586	1.161[0.679; 1.985]
No	81(21.5%)	94(24.9%)	0^a^	.	.
Unboiled water					
Yes	134(35.5%)	169(44.8%)	-0.615	0.100	0.540[0.260; 1.125]
No	17(4.5%)	57(15.1%)	0^a^	.	.
Bottled water					
Yes	76(20.2%)	115(30.5%)	-0.102	0.727	0.903[0.510; 1.599]
No	75(19.9%)	111(29.4%)	0^a^	.	.
Swimming					
Yes	58(15.4%)	123(32.6%)	-01.016	0.066	0.362[0.123; 1.067]
No	93(24.7%)	103(27.3%)	0^a^	.	.
Warding					
Yes	54(14.3%)	109(28.9%)	0.563	0.312	1.756[0.589; 5.229]
No	97(25.7%)	117(31%)	0^a^	.	.
Level for Episodes of Fever					
Five Times and More	3(0.8%)	7(1.9%)			
Four times	4(1.1%)	2(0.5%)	0.820	0.330	2.270[0.436; 11.809]
Three times	12(3.2%)	10(2.7%)	0.452	0.576	1.571[0.323; 7.633]
Two times	20(5.3%)	29(7.7%)	0.992	0.252	2.696[0.494; 14.721]
One time	20(5.3%)	25(6.6%)	1.411	0.240	4.099[0.389; 43.200]
None	92(24.4%)	153(40.6%)	0^a^	.	.
Jaundice in last year					
Yes	8(2.1%)	16(4.2%)	-0.716	0.173	0.488[0.174; 1.368]
No	143(37.9%)	210(55.7%)	0^a^	.	.
Diagnosed malaria in last year					
Yes	50(13.3%)	57(15.1%)	0.183	0.638	1.200[0.560; 2.572]
No	101(26.8%)	169(44.8%)	0^a^	.	.

^a^. This parameter is set to zero because it is redundant.

Source: Field Survey,2016

Using MAT as the gold standard, we calculated ELISA IgG to have a sensitivity of 65.4% and a specificity of 95.4%. For ELISA IgM we found a sensitivity of 8.8% and a specificity of 97.5%, with a PPV of 66.7% and an NPV 65.5%. When compared with the gold standard MAT, we found both ELISA IgG and IgM to have high specificity (95.4% and 97.5%, respectively), but very low sensitivity (65.4% and 8.8%, respectively). For that reason, for this study, we used the total number of positives, combining all the assays as a measure to increase the power to determine prevalence.

### Demographic features associated with leptospirosis exposure

The results of the analysis as depicted by the (Table 3) showed that male have higher prevalence of leptospira (34.7%) compared to female (5.3%). The same behaviour also occurs for the case of negative serology. With regard to the district of origin, Huye district has had many people in both cases (negative and positive). The assessment in occupations, we have significant affecting factors like livestock farmers, cattle farmers as well as Slaughterhouse workers. This is said due to the fact that they p-values are less that standards level of significance (5%).

**Table 3:** Multivariate Analysis for the likelihood of Demographic characteristics of asymptomatic Rwandan adult participants.

	Prevalence out total number of participants (n = 377)		B	p-value	OR [95% C.I]
	Positive (40.1%)	Negative (59.9%)			
**Intercept**			-1.722	0.000	
**Sex of Participants**					
Male	131(34.7%)	185(49.1%)	0.513	0.099	1.670 [0.908; 3.071]
Female	20(5.3%)	41(10.9%)	0^a^	.	.
**District**					
Gisagara	45(11.9%)	99(26.3%)	-0.127	0.687	0.881 [0.475; 1.632]
Huye	106(28.1%)	127(33.7%)	0^a^	.	.
**Occupation**					
Slaughterhouse workers	27(7.2%)	41(10.9%)	0.879	0.036	2.409 [1.061; 5.469]
Livestock farmer	36(9.5%)	38(10.1%)	1.392	0.002	4.021 [1.636; 9.886]
Cattle farmer	52(13.8%)	46(12.2%)	1.553	0.001	4.727 [1.863; 11.997]
Raising fish	21(5.6%)	48(12.7%)	0.538	0.207	1.713 [0.742; 3.953]
Student	15(4%)	53(14.1%)	0^a^	.	.

^a^. This parameter is set to zero because it is redundant.

Source: Field Survey,2016

Regarding animal exposures (Table 4), the vast majority of subjects with leptospiral antibodies reported contact with rats equivalent to 137(36.3%), Cow Exposure at 131(34.7%) among others. The results of both prevalence ratio and odd ratio portrays that the proportion of participants with positive leptospira serology have higher fold greater than those with negative leptospira in case they were exposed to rats. Beside rats, Dog Exposure and Cow Exposure also were found to have high fold of causing leptospira compared to other animals. The analysis also found that it would be a serious mistake to ignore the effect of all categories of animals when their autocorrelation.

**Table 4:** Multivariate Analysis for the likelihood of effect of animal exposure and *Leptospira* infection among asymptomatic adults.

	Prevalence out total number of participants (n = 377)		Coefficient	p-value	OR [95% C.I]
	Positive (40.1%)	Negative (59.9%)			
Intercept Rat Exposure			-1.183	0.000	
Yes	137(36.3%)	182(48.3%)	0.777	0.113	2.175 [0.833; 5.681]
No	14(3.7%)	44(11.7%)	0^a^	.	.
Dog Exposure					
Yes	124(32.9%)	166(44%)	0.038	0.919	1.039 [0.497; 2.170]
No	27(7.2%)	60(15.9%)	0^a^	.	.
Pig Exposure					
Yes	112(29.7%)	153(40.6%)	-0.065	0.826	0.937 [0.523; 1.677]
No	39(10.3%)	73(19.4%)	0^a^	.	.
Cow Exposure					
Yes	130(34.5%)	177(46.9%)	0.154	0.692	1.166 [0.545; 2.497]
No	21(5.6%)	49(13%)	0^a^	.	.
Goats Exposure					
Yes	131(34.7%)	178(46.9%)	0.000	1.000	1.000 [0.414; 2.416]
No	21(5.6%)	48(12.7%)	0^a^	.	.

^a^. This parameter is set to zero because it is redundant

Source: Field Survey,2016

## Discussion

To our knowledge, this is the first published data of leptospiral antibodies prevalence and leptospirosis in Rwanda. In our study of 377 asymptomatic people from two rural districts, we found a high prevalence of leptospiral antibodies. This may suggest a high prevalence of prior leptospirosis exposure, compared with studies in nearby countries. Nearly half of all subjects showed evidence of current or prior infection.

It is unclear why the prevalence we found in rural Rwanda, confirmed by multiple assays, is higher than nearby countries. The possible reasons for the observed higher prevalence in Rwanda could be the wider inclusion of the live antigens in the MAT which included 30 serovars including the most recommended representatives of serogroup Icterohaemorhagiae which is reported to be wide spread in the East African region [17] which shows that inclusion of local serovars especially serovar Sokoine belonging to serogroup Icterohaemorrhagiae increases seropositivity detection by 10 fold. In this study serogroup Icterohaemorrhagiae was well represented by serovars Icterohaemorrhagiae (RGA), Copenhageni (M 20 and L1 130).

This corroborate well with previous reported widespread of members of this serogroup in broad range of host species in this region [17]. Recent studies in countries near Rwanda have found a much lower leptospirosis prevalence, between 7 and 16% [7.11]. Further from Rwanda, however, there are data showing higher rates of leptospirosis prevalence, similar to what we found in this study. For example, a study of 105 healthy asymptomatic subjects in Somalia found that 37% were positive for leptospirosis antibodies [18]. Serogroup Icterohaemorrhagiae was the most prevalent among the positive samples determined by MAT at 20.8%(n = 78/377, 20.8%) which was higher than studies conducted in Mwanza [11] and Morogoro [17], Tanzania which also showed predominance of serogroup of Icterohaemorrhagiae.

Possible explanations for variation in prevalence include the geography of which Rwanda has one of the highest population densities in Africa with 415 inhabitants per square kilometer [14] and climate of Rwanda where two main rain seasons are observed including the short rains in October till mid-march and heavy downpours almost daily, alternating with sunny weather, as well as differences in cultural habits or occupational and farming practices in the region.

Our findings in Rwanda suggest that leptospirosis may be responsible for undiagnosed illness in the region. Six individuals met criteria for a recent acute infection based on a MAT titer ≥ 1:800, despite the fact that they reported no symptoms and absence of fever was objectively documented. Of these, 4 were crop farmers, which is consistent with our finding among the larger population that crop farming increases the odds of leptospirosis infection and may be subject to continuous exposure to the bacteria. In addition, 4 of these 6 subjects reported both a diagnosis of malaria and having had a subjective fever in the past year. This suggests that in some instances, leptospirosis infection is occurring undiagnosed in the population, which is consistent with what has been found in prior studies [19,20].

It is possible that, in some cases, treating presumed malaria will not be beneficial if the febrile illness is due to leptospirosis. Other than being mistaken for malaria, leptospirosis can also mimic other important tropical viral illnesses, such as dengue and influenza [21]. This is particularly relevant, because unlike these other diseases, leptospirosis is treatable with easily available antibiotics such as doxycycline or penicillin. Further research is needed to differentiate febrile illness caused by leptospirosis from other tropical illnesses in Rwanda [22,23].

We collected data from two rural sites, and found a significantly higher leptospiral antibodies in Gisagara district compared with Huye district which was represented mainly by university students. The most likely reason is that the University is located in Huye, and all students were enrolled at that location. Students were found to have a significantly lower leptospiral antibodies prevalence than other occupational groups, so the regional variation in prevalence is most likely a marker for the occupational differences at those sites. Future studies should collect data from more locations around Rwanda, in order to confirm the factors that explain this regional variation in leptospirosis prevalence.

Prior research elsewhere has established pigs, rats and dogs as common animal reservoirs for leptospirosis [8,13,24,25], but the organism is also found in many domestic livestock mammals, including pigs [9]. The fact that nearly all subjects in our study had contact with rats is consistent with prior research suggesting that rats may be an important reservoir host for leptospirosis transmission in Rwanda. However, our data also show that rats are not the reservoir for leptospirosis.

We found one occupation that increased both odds of leptospirosis and prevalence ratio, which was being a crop farmer, and one occupation that was found to be less exposed against leptospirosis, which was being a student with lower age categories since as years go up people tend to be more exposed and turn positive for leptospiral antibodies. The high prevalence of leptospirosis in crop farmers, compared with other occupations such as raising fish, further supports the hypothesis that rats and domestic livestock are the major host transmitters for leptospirosis in Rwanda. These findings, if confirmed in larger, more rigorous studies, could have important implications for leptospirosis control initiatives in Rwanda.

Of the six exposures related to consumption of milk and water, we found one (drinking unboiled water) that increased the odds of leptospirosis in univariate analysis, and we found two protective factors (drinking pasteurized milk and drinking boiled water). None of these exposures was found to be independently associated with leptospirosis in multivariate analysis. These findings are consistent with prior research [11,26,27], which has established that a drinking supply contaminated by animal urine can be a common means of leptospirosis transmission.

The major limitation of our study is the potential for selection bias. We did not randomly select subjects for this study, but rather chose them based on their occupation. Because of this, our results cannot be generalized to the entire Rwandan population. Our data was collected in two rural areas of Rwanda, and the prevalence we report is not externally generalizable to other regions of Africa or the world.

Since four of the occupations we chose to sample purposively are known to have high exposure to Leptospirosis, the prevalence among individuals in these categories (slaughterhouse worker, cattle farmer, crop farmer, and fish farmer) considered to be higher than other Rwandans. Thus, the occupations we selected for sampling, when added together, represent a majority of the occupational work that Rwandans living in rural areas are engaged Wirth in. We included University students as our baseline population. As we anticipated, the students had the least exposure levels to leptospirosis. However, 15 of the 68 equivalent to 1 of the 5 students was found to have antibodies against *Leptospira* species, which was higher than we expected.

One explanation for this finding may be that University students reported similar rates of animal exposure as other occupations (40–60%), something we did not anticipate. It may be that students are traveling home on weekends and holidays, and they have contact with animals in those settings. The presence of leptospirosis among students may have lowered the power of the study to detect differences among the occupational groups.

In conclusion, we found that leptospirosis infection is highly prevalent in selected rural Rwanda. Risk factors for leptospirosis positivity by univariate analysis included drinking unboiled water, swimming, exposure to rats, and working as a crop farmer. Protective factors included drinking pasteurized milk, drinking boiled water, and being a student with lower age categories in the age range of 21–31 years old. Although none of these exposures, however, were found to be independently associated with leptospirosis infection, this study addresses the gap of information for leptospirosis in Rwanda, highlighting the main aspects that contribute to the propagation of this important neglected disease in this population.

Compared to the neighboring countries, the observations of leptospiral antibodies in 5 occupation groups: two groups show lower prevalence, for slaughterhouse worker is 7.2% while in Tanzania is reported to 10% [12] and Crop farmers is 13.8% while recently reported prevalence of antibodies in similar occupation in Tanzania is 18.4% [13], Cattle farmers in this study (9.5%) that appears to lower than 29.9% found in Tanzania among asymptomatic pastoralists [28]. The prevalence of antibodies in fish farmers in this study (5.6%) appears to be the lower than what was recently reported in Kagera region of Tanzania (14.8%) [13].

Further studies are needed to understand the epidemiology and impact of this disease in Rwanda. The collection and isolation of leptospires would allow researchers to gain greater insight into the spectrum of local Leptospira serovars in Rwanda. Larger studies would also facilitate greater awareness of leptospirosis among Rwandan clinical practioners and health authorities, and assist with identifying methods of prevention.

## Supporting information

S1 Table Sample codes and test results. (XLSX)

S1 Data Database of participants `information. (SAV)

S1 Text Legends and Acronyms. (DOCX)

S1 Strobe checklist STROBE Observational Study Checklist. (DOCX)
