# Supplementary material for: Sero-prevalence of anti-Leptospira antibodies and associated risk factors in rural Rwanda: A cross-sectional study
Source: PLoS Negl Trop Dis. 2021 Dec 7;15(12):e0009708. doi: 10.1371/journal.pntd.0009708 (PMC8683035; doi:10.1371/journal.pntd.0009708)
Supplement: S1 Text — (DOCX) [file pntd.0009708.s003.docx]

**LEGENDS AND ACRONYMS**

CT: State of Connecticut, United States

BA: State of Bahia, Brazil

NY: State of New York, United States

UT: State of Utah, United States

ELISA: The Enzyme-Linked Immunosorbent Assay

MAT: Microscopic Agglutination Test

CI: Confidence intervals

Spp: Species

OR: Odds Ratio

B: Coefficient

0^b^:

US: United States

LPHS: Leptospiral Pulmonary Hemorrhage Syndrome

IgG: Immunoglobulin G

IgM: Immunoglobulin M

EMJH: Ellinghausen-McCullough-Johnson Harris

OD: Optical Density

SPSS: Statistical Package for the Social Sciences

PPV: Positive Predictive Value

NPV: Negative Predictive Value

RGA: Rabbit Antisera Reactions

NISR: National Institute of Statistics of Rwanda.
