# Supplementary material for: Sero-prevalence of anti-Leptospira antibodies and associated risk factors in rural Rwanda: A cross-sectional study
Source: PLoS Negl Trop Dis. 2021 Dec 7;15(12):e0009708. doi: 10.1371/journal.pntd.0009708 (PMC8683035; doi:10.1371/journal.pntd.0009708)
Supplement: S1 Strobe checklist — (DOCX) [file pntd.0009708.s004.docx]

STROBE Observational Study Checklist

**Title and Abstract**

**1 a :** Sero-prevalence of anti-Leptospira antibodies and associated risk factors in rural Rwanda: a cross-sectional study

**b :** Lines 10-18 : We performed a cross-sectional survey of asymptomatic adults recruited from five occupational categories. Serum samples were tested using ELISA and Microscopic Agglutination Test (MAT). We found that 40.1% (151/377) of asymptomatic adults had been exposed to *Leptospira* spp. Almost 91% of positive subjects reported contact with rats (137/151) compared with 80.5% of negative subjects (182/226, OR 2.37, CI 1.25-4.49). Furthermore, being a crop farmer was significantly associated with leptospirosis (OR 2.06, CI 1.29 – 3.28). We identified 6 asymptomatic subjects (1.6%) who met criteria for acute infection.

**Introduction**

**2: Background/rationale**

Lines 67-76: Prevalence and incidence data from Africa is still scarce. In Sub-Saharan Africa, there are a number of factors that put the population at increased risk of leptospirosis infection, including urban population density, poor infrastructure to manage flooding, and a large proportion of the population lacking footwear.^11^ In November 2005, a cross-sectional study conducted in Tanzania, which borders Rwanda, showed a seroprevalence of 15% in 199 healthy participants.^12^ A study done in two parts of Kenya, which is in the same region of East Africa as Rwanda, found a prevalence of 16.9% among 130 asymptomatic adults in the coast province, and 7.4% among 353 healthy people in Nyanza province (near Lake Victoria).

**3: Objectives**

We have added the following (lines 77-84) to our manuscript:

**Specific Objective 1:** To determine the prevalence of *Leptospira* in the Southern region of Rwanda.

**Specific Objective 2:** To determine which exposures and risk factors are associated with Leptospirosis infection in the Southern region of Rwanda.

**Hypothesis 1**: The asymptomatic population of Southern Rwanda has a moderate to high prevalence of prior infection with leptospirosis.

**Hypothesis 2:** Among asymptomatic subjects in Southern Rwanda, previous infection with Leptospirosis correlates with previously established risk factors.

**Methods**

**4: Study design**

In the first sentence of the second paragraph of the Methods section (lines 104-108) we present key elements of the study design: “We performed a prospective cross-sectional study of asymptomatic individuals of working age (≥ 21 years), from Gisagara and Huye districts in the Southern Province of Rwanda.”

**5: Setting**

Lines 104-112: During two weeks in January 2016, we recruited subjects from 5 occupations. After consent was obtained and demographic data collected, whole blood was drawn from each subject and transported immediately to the Serology Unit at the University Hospital of Butare.

**6: Participants**

Lines 99-103: To be eligible for inclusion in the study, subjects had to be 21 years of age or older and have no current medical complaints. We took a set of vital signs for every potential subject, and candidates were excluded if they were found to have a temperature of 38.0 C or above.

**7 : Variables**

Outcomes were defined in the Methods section under the sub-heading Case Definition (Lines 138-142): “Each sample was tested with all three assays: MAT and ELISA for IgG and IgM anti-*Leptospira* antibodies. Subjects were classified as positive if they met any of the three following criteria: 1) ELISA IgG OD > 1.0, 2) ELISA IgM OD > 1.0, and/or 3) MAT > 1:100.”

Exposures are described briefly in the manuscript text (Lines 202-208), and every exposure is listed in detail in Table 2 and Table 3.

**8: Data sources/measurement**

Under the sub-heading “Blood sampling and serological screening” (Lines 109-125) we describe how samples were obtained, stored, processed and analyzed, including details about the equipment manufacturer used.

**9: Bias**

In the Methods section we state that we collected data at the site where participants were making their daily activity, usually work. We state that “We evaluated all potential subjects equally and included every subject who met eligibility.” (line 109) Beyond this, we had no formal structures in place to address potential sources of bias.

In the Discussion section of our manuscript we address the question of selection bias (Lines 296-308) and acknowledge it as a limitation with respect to the generalizability of our results.

**10: Study Size**

This is the first study of Leptospirosis in Rwanda. Our goal was to provide some preliminary understanding of prevalence and risk factors to help guide larger, more statistically rigorous work. Study size was arrived at based on the number of subjects that it was logistically feasible for us to recruit and enroll given our limited resources.

**11: Quantitative variables**

The most important quantitative variables in our study were the two ELISA tests for IgG and IgM, and the MAT test. These three variables were used to determine whether or not each subject had been exposed to Leptospirosis, and the specific cut-offs we used to create these groupings are described in lines 140-142. Our cut-offs were chosen consistent with the assay manufacturer’s guidelines and with prior Leptospirosis studies to maximize the accuracy of our results.

**12: Statistical methods**

**a)** Correlations were performed between overall leptospirosis status and every demographic factor. For continuous variables such as age we used the student’s t-test (independent sample t test), and for categorical variables we used the Chi Square test (Lines 144-151). Confidence intervals for risk ratios were calculated by exact methods. We also performed binary logistic regression using the Statistical Package for Social Science (20 IBM SPSS STASTICS).

**b)** We did not perform any subgroup analysis. We performed both univariate and multivariate analysis of both animal and non-animal exposures to assess for interactions between risk factors, with the results reported in Tables 2 and 3.

**c)** The methodology of this study involved a single interaction between researcher and subject. Blood was drawn and demographic data collected for all patients. There were no instances of missing data, and due to the cross-sectional methodology there was no risk of subjects being lost to follow-up.

**d)** This was a small, two-site study that collected 377 specimens. It does not purport to accurately reflect leptospirosis prevalence throughout Rwanda, which we acknowledge at several points in the manuscript (lines 281-283; lines 293-295; lines 305-307). We used no analytical methods taking account of sampling strategy.

**e)** We performed no sensitivity analyses for this small cross-sectional prevalence study.

**Results**

**13: Participants**

**a)** Due to cultural and logistical limitations at the data collection site (often the place of employment in rural Rwanda), we were not able to collect data on subjects who were eligible to participate but chose not to.

**b)** All subjects who enrolled in the study completed it and were analyzed. There was no missing data.

**c)** The methodology of this study involved a single interaction between researcher and subject. We did not have data on individuals who might have been eligible but chose not to participate. Furthermore, we had no missing data or lost-to-follow up data. Therefore, we chose not to include a flow diagram.

**14: Descriptive data**

**a)** We provide data on characteristics of study participants in lines 190-198, with additional details in Table 1. We provide information on animal exposures in lines 211-218 and Table 2. We provide information on water and milk exposures in lines 229-238 and Table 3.

**b)**  We had no missing data for subjects enrolled in this study.

**15: Outcome data**

Our outcome in this study was Leptospirosis antibody positivity. We report overall numbers of antibody positivity under the subheading “Serology” in lines 161-170.

**16: Main results**

**a)** We did not adjust for confounding in this study.

**b)** Under the sub-heading “Case Definition” we describe how we converted the continuous variable from the MAT and ELISA assay into a categorical variable (lines 140-142).

**c)** We chose to report odds ratios in this study and do not use relative risk in either the manuscript text or any of our tables.

**17: Other analyses**

We did not do any sub-group analysis, sensitivity analysis, or analysis of interactions.

**Discussion**

**18: Key results**

*Specific Objective 1:* *Establish prevalence of leptospirosis in Rwanda.* In the first paragraph of our Discussion section (lines 250-257) we reference the prevalence of leptospirosis, which we characterize as high relative to surrounding countries.

*Specific Objective 2: Correlate leptospirosis positivity with established risk factors.* In the 4^th^ and 5^th^ paragraphs of our Discussion section (lines 285-303) we analyze our findings with regard to which exposures (animals, milk, water, etc) were found to correlate with increased leptospirosis infection.

**19: Limitations**

In the 6^th^ paragraph of our Discussion section (lines 305-317) we acknowledge the potential for selection bias due to the fact that we did not randomly sample subjects but chose them based on occupation. In our earlier manuscript we did not specifically address the direction and magnitude of bias. We have added the following (lines 314-317): “Our findings may overestimate the true prevalence of leptospirosis in Rwanda. However, since the occupations we selected for sampling, when added together, represent a majority of the occupational work that Rwandans living in rural areas engage in, we believe that this overestimation is limited to 10% or less.”

**20 Interpretation**

In the opening and closing paragraphs of our Discussion section, we provide an overall cautious interpretation of our findings that references our objectives (251-253 and lines 328-330), results from similar studies (lines 251-257), and the types of analyses we did (lines 330-335).

**21 Generalisability:**

We have added the following sentence (lines 307-309): “Our data was collected in two rural areas of Rwanda, and the prevalence we report is not externally generalizable to other regions of Africa or the world.”
